# Man-Specific, GalNAc/T/Tn-Specific and Neu5Ac-Specific Seaweed Lectins as Glycan Probes for the SARS-CoV-2 (COVID-19) Coronavirus

**DOI:** 10.3390/md18110543

**Published:** 2020-10-29

**Authors:** Annick Barre, Els J.M. Van Damme, Mathias Simplicien, Hervé Benoist, Pierre Rougé

**Affiliations:** 1 Institut de Recherche et Développement, Faculté de Pharmacie, UMR 152 PharmaDev, Université Paul Sabatier, 35 Chemin des Maraîchers, 31062 Toulouse, France; annick.barre@univ-tlse3.fr (A.B.); simplicien.mathias@gmail.com (M.S.); herve.benoist@ird.fr (H.B.); 2 Department of Biotechnology, Faculty of Bioscience Engineering, Ghent University, Coupure links 653, B-9000 Ghent, Belgium; ElsJM.VanDamme@UGent.be

**Keywords:** seaweed lectins, red algae, mannose-specific lectins, *N*-acetylgalactosamine-specific lectins, T/Tn-specific lectins, griffithsin, SARS-CoV-2, COVID-19, *N*-glycosylation, *O*-glycosylation, high-mannose glycans, glycan probes

## Abstract

Seaweed lectins, especially high-mannose-specific lectins from red algae, have been identified as potential antiviral agents that are capable of blocking the replication of various enveloped viruses like influenza virus, herpes virus, and HIV-1 in vitro. Their antiviral activity depends on the recognition of glycoprotein receptors on the surface of sensitive host cells—in particular, hemagglutinin for influenza virus or gp120 for HIV-1, which in turn triggers fusion events, allowing the entry of the viral genome into the cells and its subsequent replication. The diversity of glycans present on the S-glycoproteins forming the spikes covering the SARS-CoV-2 envelope, essentially complex type *N*-glycans and high-mannose type *N*-glycans, suggests that high-mannose-specific seaweed lectins are particularly well adapted as glycan probes for coronaviruses. This review presents a detailed study of the carbohydrate-binding specificity of high-mannose-specific seaweed lectins, demonstrating their potential to be used as specific glycan probes for coronaviruses, as well as the biomedical interest for both the detection and immobilization of SARS-CoV-2 to avoid shedding of the virus into the environment. The use of these seaweed lectins as replication blockers for SARS-CoV-2 is also discussed.

## 1. Introduction

The occurrence of lectins, formerly designated as hemagglutinins due to their capacity to agglutinate red blood cells from humans and various animals, in marine seaweeds has been recognized for a long time, following the pioneering works of Boyd et al. [1] and Blunden et al. [2] on British marine algae. The carbohydrate-binding specificity of some of these seaweed lectins, especially high-mannose-specific lectins extracted and purified from red algae (Rhodophyta), has been studied in detail owing to their ability to induce apoptosis in cancer cells [3,4,5,6,7] and block, at least in vitro, the replication of various enveloped viruses such as influenza, herpes, and HIV-1 [8,9,10,11,12,13,14,15].

Griffithsin (GRFT) from the red alga *Griffithsia* sp., exhibited inhibiting properties towards HIV-1 [8]. High-mannose-specific lectins from the red algae *Kappaphycus alva*rezii (KAA-2) [10,13], *Eucheuma serra* (ESA-2) [12], and *Halimeda renschii* (HRL-40) [14] also recognized high-mannose *N*-glycans (HM-glycans) occurring at the surface of the influenza virus [10,11,12,14] and HIV-1 [8,11,13]. The high-mannose-specific lectin from the green alga *Boodlea coacta* (BCA) [11] interacts more specifically with (Manα1,2)-glycans, especially as the number of terminal branched Manα1,2 is increased. Very similar antiviral properties were identified for the cyanobacterial lectins such as OAA from *Oscillatoria agardhii* [16], cyanovirin-N (CNV-N) from *Nostoc ellipsosporum* [17,18,19,20,21], microvirin MVN from *Microcystis aeruginosa* [22,23], and scytovirin SVN from *Scytonema varium* [24]. Antiviral properties of CNV-N were reported against several viruses including HIV-1 [17], influenza virus [21], herpes virus [18,19], hepatitis C virus [20], and Ebola virus [19]. Obviously, the antiviral activity of high-mannose-specific seaweed and cyanobacterial lectins correlates with their capacity to recognize the high-mannose *N*-glycans associated to different glycoprotein receptors, e.g., hemagglutinin from influenza virus [14], or gp120 from HIV-1 [13], covering the viral envelopes.

**Figure 1:**
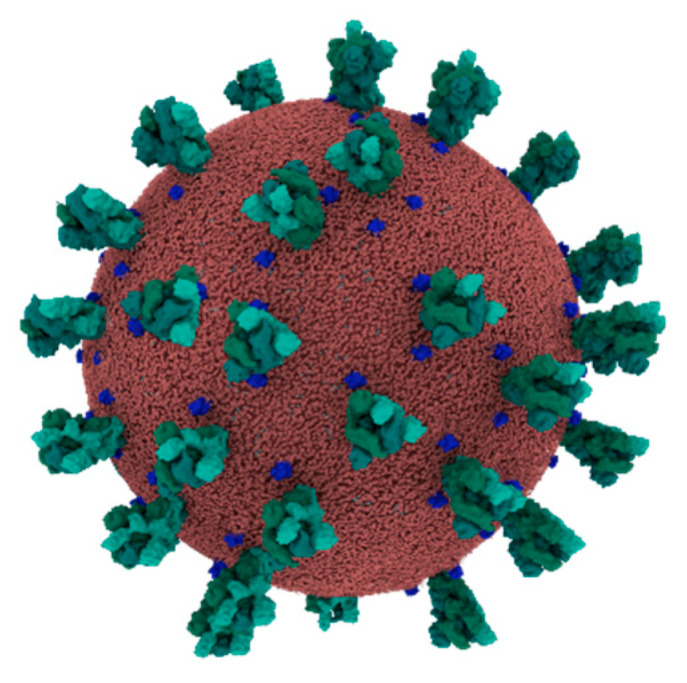
Molecular organization of the SARS-CoV-2 envelope (coronavirus credit: Maria Voigt/RCSB PDB). The spikes (colored pale green) protruding at the surface of the virus consist of homotrimers of the S-glycoprotein.

Taking into account the antiviral properties and the well known diversity of *N*-glycans decorating the spikes arrayed on the surface of the SARS-CoV-2 envelope, essentially comprised of complex *N*-glycans and high-mannose *N*-glycans [25,26], one can assume that seaweed lectins, especially high-mannose-specific seaweed lectins, can be useful tools as glycan probes for this type of coronavirus (Figure 1).

SARS-CoV-2 spikes consist of homotrimers of S-glycoproteins and play a key role in both the recognition and the subsequent membrane fusion events, resulting in the infection of the host cells [27,28]. Spikes occur in different conformations, but the closed conformation seems to occur more frequently at the surface of the viral envelope [29]. The S-glycoprotein consists of two subunits, S1 and S2, and the S1 subunit containing the receptor binding domain (RBD) is responsible for the binding to the angiotensin-converting enzyme 2 (ACE2) receptor. Motions in the RBD that are apparently pH-dependent allow the transition from a closed conformation (all RBD-down state) to an open conformation (all RBD-up state), and this transition is induced or stabilized upon binding of the RBDs to the ACE2 receptors at the surface of the host cells [30]. In turn, this recognition of ACE2 triggers the fusion of the viral envelope to the cell membrane of host cells, which allows the entry of the viral RNA genome into the cells [31]. The S2 subunit that has been proteolytically cleaved from the S1 subunit at a S1-S2 cleavage site of the S-glycoprotein, is responsible for this fusion event [32,33]. Finally, infected cells are now ready for the replication of the virus and the subsequent release of virions out of the dying host cells. This viral cycle is quite common among viruses, but SARS-CoV-2 is different from other closely related viruses in that it specifically recognizes ACE2 proteins as potential receptors, using the RBDs of the S-glycoprotein. This recognition and binding event accounts, at least in part, for the ability of SARS-CoV-2 to preferentially target host cells that are particularly rich in ACE2 protein receptors at the cell surface, such as kidney cells [34].

Due to the high number of high-mannose *N*-glycans of the S-glycoproteins forming the SARS-CoV-2 spikes [25,26,35], high-mannose-specific seaweed lectins appear as relevant glycan probes both for analytical purposes, e.g., the detection or immobilization of the virions, and for biomedical purposes, e.g., preventing the replication and fusion events of SARS-CoV-2. In this review, we present a detailed analysis of the carbohydrate-binding specificities of seaweed lectins that could be used as glycan probes for the SARS-CoV-2 coronavirus.

## 2. Seaweed Lectins of Various Carbohydrate-Binding Specificities

According to their carbohydrate-binding specificity towards simple sugars, (seaweed) lectins can been classified in five main groups of Man-specific lectins, GlcNAc-specific lectins, Gal/GalNAc-specific lectins, Fuc-specific lectins, and Sia-specific lectins. Until now, the research on seaweed lectins has focused especially on the Man-specific lectins, and these lectins have been characterized in more detail.

### 2.1. Man-Specific Seaweed Lectins

To date, a large number of seaweed lectins have been screened, but only a few lectins have been studied in detail or have been characterized at the molecular/structural level. In spite of these limitations, the amino acid sequences and some structural information have become available for some Man-specific seaweed lectins from the red algae (Rhodophyta), the yellow-green algae (Ochrophyta), and the green algae (Chlorophyta) (Table 1).

Man-specific seaweed lectins belong to a few well defined protein families which have been previously identified and characterized for the molecular organization of their protomers, especially in plants [50]:

- The GNA (*Galanthus nivalis agglutinin*)-related family of lectins consists of protomers organized in a β-prism II or β-trefoil. The red alga *Grateloupia chiangii* lectin (GCL) and the green alga *Boodlea coacta* lectin (BCA) present this type of structural organization.
- The jacalin-related family of lectins contains protomers organized in a β-prism I or β-barrel. Most of the mannose-specific lectins from red algae belong to this group of lectins, e.g., griffithsin and lectins from the genera *Gracilaria*, *Eucheuma*, *Grateloupia*, *Kappaphycus*, and *Solieria.*
- The legume lectin-related family is made of protomers organized in a β-sandwich or jelly roll fold (two β-sheets). This structural scaffold occurs in a few lectins from the genera *Hydropuntia* (red algae), *Nannochloropsis* (yellow-green algae), *Ostreococcus* (green algae), and *Porphyra* (red algae).

Man-specific seaweed lectins readily accommodate Man, oligomannosides, and high-mannose type glycan chains. In addition, most of them recognize the tri-mannosyl core Manα1,3-Manα1,6-Man occurring in both *N*-acetyllactosaminic type glycans and high-mannose type glycans.

#### 2.1.1. Man-Specific Seaweed Lectins With a β-Prism II Structure

**Figure 2:**
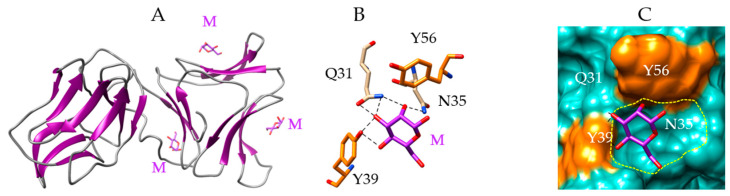
Molecular modeling of lectin from *Grateloupia chiangii*. (**A**) Lateral view of the ribbon diagram of the modeled lectin from *Grateloupia chiangii* (GCL), in complex with mannose (M, colored purple). The lectin dimer consists of the association of two differently oriented protomers exhibiting a β-trefoil fold. Man residues occupying the three CBS of the second protomer are represented. (**B**) Network of hydrogen bonds (black dashed lines) anchoring Man (M) to the amino acid residues Q31, N35, and Y39, forming the CBS-I of GCL. Aromatic residues Y39 and Y56 participating in stacking interactions with the pyranose ring of Man, are colored orange. (**C**) Molecular surface (colored slate green) at the CBS-I of GCL, forming a depression (delineated by a yellow dashed line) harboring the Man (M, colored purple) linked by a network of hydrogen bonds (black dashed lines) to Q31, N35, and Y39 residues, and stacking interactions with Y39 and Y56 residues (colored orange).

The *Grateloupia chiangii* lectin (GCL) offers a nice example of a Man-specific red alga lectin with a β-prism II structure. The β-prism II scaffold consists of three bundles of four antiparallel β-strands arranged into a flattened trefoil-shaped structure around a central pseudoaxis. The GCL lectin dimer consists of two covalently linked swapped protomers organized in a β-trefoil in such a way that both protomers become oriented almost orthogonally (Figure 2A). Each protomer contains three carbohydrate-binding sites (CBS-I, CBS-II, and CBS-III) that form a shallow depression which accommodates a Man residue via a network of six hydrogen bonds involving Q31, N35, Y39 residues, and stacking interactions between the aromatic residues Y39 and Y56 and the pyranose ring of mannose (CBS-I) (Figure 2B,C). The green algae group, *Boodlea coacta*, also contains a Man-specific lectin (BCA) with a β-prism II structure [46].

#### 2.1.2. Man-Specific Seaweed Lectins with a β-Sandwich Structure

**Figure 3:**
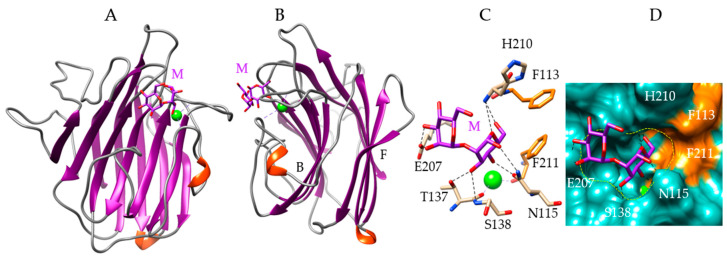
Molecular modeling of *Porphyra umbilicalis* lectin. (**A**,**B**) Back face (**A**) and lateral view (**B**) of the ribbon diagram of the modeled Porphyra umbilicalis lectin (PUL) in complex with a dimannoside Manα1,2Man (M, colored purple). The calcium ion is colored green. The front (F) and back (B) faces of the β-sandwich are indicated in B. (**C**) Network of hydrogen bonds (black dashed lines) anchoring the dimannoside Manα1,2Man (M) to the amino acid residues forming the CBS of the PUL. Aromatic residues F113 and F211 interacting with the dimannoside by stacking interactions, are colored orange. (**D**) Molecular surface (colored slate green) at the CBS of PUL forming a depression (delineated by a yellow dashed line) harboring the dimannoside (M, colored purple) linked by a network of hydrogen bonds (black dashed lines) to N115, T137, S138, E207, and H210 residues, and stacking interactions with F113 and F211 residues (colored orange). The calcium ion is colored green.

The *Porphyra umbilicalis* lectin (PUL) illustrates the β-sandwich organization of the lectin protomer, resulting from the covalent superposition of two strands of β-sheets connected by more or less extended loops, forming the front and back faces of the β-sandwich, respectively (Figure 3A,B). This type of structural organization is very common in plant lectins belonging to the Fabaceae or Leguminosae family, such as Con A [51], pea [52], and lentil [53] lectins. Animal lectins, such as the LMAN1/ERGIC-53 protein [54] or the VIP36 protein [55], also possess a β-sandwich structure. A few amino acid residues belonging to the loops connecting both strands of β-sheets in the β-sandwich structure (Figure 3B), form the CBS which accommodates Man and dimannosides via a network of hydrogen bonds with residues N115, T137, S138, E207 and H210, and stacking interactions with the aromatic residues F211 and F213 (Figure 3C). The CBS appears as a shallow depression at the molecular surface of the lectin, in which the Man units become anchored (Figure 3D). Two other Man-specific lectins with a β-sandwich structure have been identified in the green-yellow alga *Nannochloropsis gaditana* (BU14), and in the green alga *Ostreococcus tauri* (OtL) [49] (Table 1).

#### 2.1.3. Man-Specific Seaweed Lectins with a β-Prism I Structure

**Figure 4:**
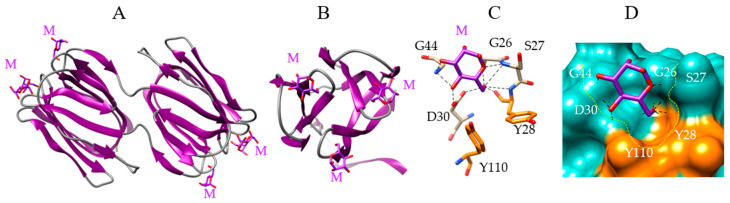
Molecular modeling of griffithsin. (**A**,**B**) Lateral (**A**) and front view (**B**) of the ribbon diagram of the domain-swapped griffithsin, in complex with mannose (M) (PDB code 2GUD). (**C**) Network of hydrogen bonds (black dashed lines) anchoring mannose (M) to the amino acid residues forming the CBS of griffithsin. Aromatic residues Y28 and Y110 participating in stacking interactions with the pyranose ring of Man, are colored orange. (**D**) Molecular surface (colored slate green) at the CBS of griffithsin forming a depression (delineated by a yellow dashed line) harboring the Man (M, colored purple) linked by a network of hydrogen bonds (black dashed lines) to G26, S27, Y28, D30, and G44 residues, and stacking interactions with Y28 and Y110 residues (colored orange).

Griffithsin, the Man-specific jacalin-related lectin firstly isolated and characterized from the red alga *Griffithsia* sp., is the prototype of a group of giffithsin-like lectins occurring in red algae, especially in the genera *Agardhiella*, *Eucheuma*, *Gracilaria*, *Kappaphycus*, *Meristiella*, *Meristotheca*, and *Solieria* (Table 1). The domain-swapped griffithsin consists of two non-covalently associated domains exhibiting a β-barrel structure built up from three four-stranded antiparallel β-sheets (Figure 4A). Loops connecting the β-sheets form three CBS at the top of the β-barrel (Figure 3A,B). A front view of the β-barrel shows that the CBS adopt a triangular pattern at the top of each protomer (Figure 4D).

Each of the three CBS accommodates Man via a network of nine hydrogen bonds with G26, S27, Y28, D30, and G44 residues (for the 1st CBS), and two additional stacking interactions with aromatic residues Y28 and Y110 (Figure 4C). The CBS form a shallow depression largely open at the molecular surface of the lectin, in which Man is anchored by hydrogen bonds and hydrophobic interactions (Figure 4D).

**Figure 5:**
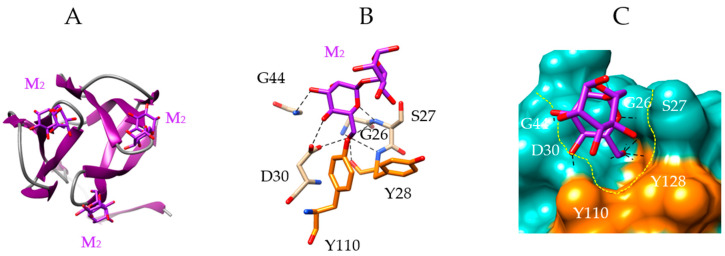
Molecular modeling of griffithsin. (**A**) Front view of the ribbon diagram of a domain of griffithsin, in complex with dimannoside Manα1,6Man (M, colored purple) (PDB code 2HYQ). (**B**) Network of hydrogen bonds (black dashed lines) anchoring the dimannoside (M) to the amino acid residues forming the CBS of griffithsin. Aromatic residues Y28 and Y110 participating in stacking interactions with the dimannoside, are colored orange. (**C**) Molecular surface (colored slate green) at the CBS of griffithsin forming a depression (delineated by a yellow dashed line) harboring the dimannoside (M, colored purple) linked by a network of hydrogen bonds (black dashed lines) to G26, S27, Y28, D30 and G44 residues, and stacking interactions with Y28 and Y110 residues (colored orange). Note the absence of contact between the second Man residue of the dimannoside and the CBS of griffithsin.

The CBS of griffithsin also accommodates dimannosides, e.g., Manα1,6Man, via a similar network of 8 hydrogen bonds and stacking interactions with the Y28 and Y110 residues, but the second Man unit located at the reducing end of the disaccharide does not participate in the interaction (Figure 5A,B). A front view of the CBS shows the absence of contact of the second Man of the dimannoside with the lectin (Figure 5C).

**Figure 6:**
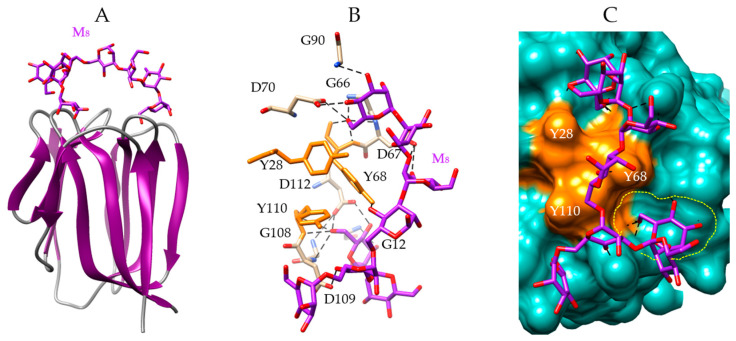
Anchoring of Man_8_ to griffithsin. (**A**) Lateral view of the ribbon diagram of griffithsin in complex with a high-mannose branched chain (M_8_, colored purple) (PDB code 3LL2). (**B**) Network of hydrogen bonds (black dashed lines) anchoring M_8_ to the amino acid residues forming CBS-I (G12), CBS-II (G66, D67, Y68, D70), and CBS-III (G90, G108, D109, Y110, D112) of griffithsin. Aromatic residues Y28, Y68 and Y110 participating in stacking interactions with the M_8_, are colored orange. (**C**) Molecular surface (colored slate green) at the CBS-II and CBS-III of griffithsin, forming a depression (delineated by a yellow dashed line) harboring M_8_ (M_8_, colored purple) linked by a network of hydrogen bonds (black dashed lines) to G12, G66, D67, Y68, D70, G90, G108, D109, and D112 residues, and stacking interactions with Y28, Y68, and Y110 residues (colored orange).

According to the triangular disposition of the three CBS at the top of the griffithsin protomers, both CBS can participate in the binding of a complex octasaccharide (Man_8_) to the lectin (Figure 6A). The Man8 interact with the lectin via a network of 18 hydrogen bonds with residues G12 (first CBS), G66, D67, Y68, D70 (second CBS), and G90, G108, D109, Y110, and D112 (third CBS), together with stacking interactions with aromatic residues Y28 (first CBS), Y68 (second CBS), and Y110 (third CBS) (Figure 6B). A front view of the protomer linked to Man_8_ oligosaccharide, shows that the second and third CBS of griffithsin play a major role in the interaction with the octamannoside (Figure 6C).

**Figure 7:**
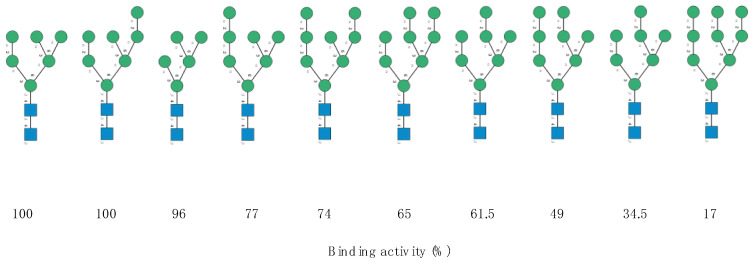
Structure of the high-mannose type *N*-glycans assayed by Sato et al. (2011) to measure the oligosaccharide-binding specificity of KAA-2 from the red alga *Kappaphycus avalvarezii*. The high-mannose *N*-glycans are aligned according to their decreasing binding activity (expressed as %) towards KAA-2 (adapted from [10]). Symbols used to represent *N*-glycans: blue squares: *N*-acetylglucosamine, green circles: mannose.

The carbohydrate-binding specificity of another Man-specific lectin with a β-prism I structure from the red alga *Kappaphycus alvarezii* (KAA-2) towards high-mannose glycans, has been studied in detail by Sato et al. [10]. The high-mannose glycan-binding activity of the lectin was measured towards a series of bi- and tri-antennary branched high-mannose glycans (Figure 7).

**Figure 8:**
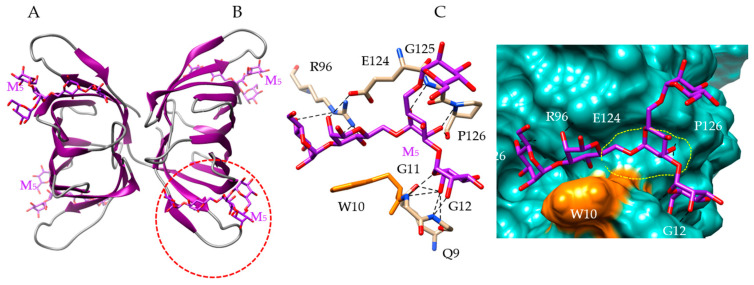
Molecular modeling of *Kappaphycus alvarezii lectin*. (**A**) Lateral view of the ribbon diagram of the modeled KAA-2 from *Kappaphycus alvarezii*, in complex with a pentamannoside chain (M_5_, colored purple). (**B**) Network of hydrogen bonds (black dashed lines) anchoring M_5_ to the amino acid residues Q9, G11, G12, R96, E124, G125 and P126 forming the CBS (red dashed circle) of KAA-1. The aromatic residue W10 which also participates in stacking interaction with M_5_, is colored orange. (**C**) Molecular surface (colored slate green) at the CBS of KAA-2, forming a large depression (delineated by a yellow dashed line) harboring M_5_ (M_5_, colored purple) linked by a network of hydrogen bonds (black dashed lines) to Q9, G11, G12, R96, E124, G125 and P126 residues, and a stacking interaction with W10 residue (colored orange).

Docking of a pentamannoside (Man_5_) to the modeled KAA-2 lectin, showed the existence of four CBS located at both ends of the β-barrel forming each protomer of the KAA-2 dimer (Figure 8A). The CBS accommodates the pentamannoside via a network of 14 hydrogen bonds with Q9, G11, G12, R96, E124, G125 and P126 residues, completed by an additional stacking interaction with the aromatic W10 residue (Figure 8B,C). All the Man units of M_5_ participate in the binding to the lectin.

Mannose-specific lectins closely-related to griffithsin, which similarly accommodate Man and oligomannosides, have been characterized in blue green algae (cyanobacteria), e.g., cyanovirin-N (CV-N) from the cyanobacterium *Nostoc ellipsosporum* [17], microvirin MVN from *Microcystis aeruginosa* [22,23], scytovirin SVN from *Scytonema varium* [24], and the *Oscillatoria agardhii* agglutinin OAA [16]. All of these cyanobacterial lectin domains also consist of a β-barrel structure [9].

### 2.2. Seaweed Lectins with GalNAc/T/Tn- and Neu5Ac-Specificity

Other seaweed lectins display a quite different binding-specificity towards GalNAc and T/Tn antigen (Table 2) and towards Neu5Ac/sialic acid ending *N*-glycans of the *N*-acetyllactosaminic type (Table 3). However, at present no sequence and structural information is available to get an insight into the molecular aspects of their carbohydrate-binding specificity.

## 3. Structural Organization and Glycosylation of S Glycoproteins Forming the Spikes of SARS-CoV-2 Virus

The spikes arrayed on the surface of SARS-CoV-2, each result from the non-covalent association of three similar S-glycoproteins in an homotrimer structural scaffold that protrudes outside the virus surface (Figure 1).

The spike S-glycoprotein consists of a single polypeptide chain of 1273 amino acids (140 kDa), containing 22 potential *N*-glycosylation sites 17NLT, 61NVT, 74NGT, 122NAT, 149NKS, 165NCT, 234NNIT, 282NNGT, 331NIT, 343NAT, 603NTS, 616NCT, 657NNS, 709NNS, 717NFT, 801NFS, 1074NFT, 1098NGT, 1134NNT, 1158NHT, 1173NAS, 1194NES (seven potential *N*-glycosylation sites 17NLT, 603NTS, 657NNS, 1134NNNT, 1158NHT, 1173NAS and 1194NES, are apparently not glycosylated) and three *O*-glycosylation sites T323, S325 and T678 are actually glycosylated [25,26,35].

As shown below the complete amino acid sequence of the S glycoprotein of SARS-CoV-2 is made of two S1 and S2 subunits. The RBD of subunit S1 is highlighted in green and the S1/S2 cleavage site for cathepsin and serine protease TMPRSS2 is highlighted in red. All the *N*-glycosylation sites NXT/NXS are highlighted in dark blue and the *O*-glycosylation sites are shown in bold letters highlighted in yellow:

VNLTTRTQLPPAYTNSFTRGVYYPDKVFRSSVLHSTQDLFLPFFSNVTWFHAIHVSGTNGTKRFDNPVLPFNDGVYFASTEKSNIIRGWIFGTTLDSKTQSLLIVNNATNVVIKVCEFQFCNDPFLGVYYHKNNKSWMESEFRVYSSANNCTFEYVSQPFLMDLEGKQGNFKNLREFVFKNIDGYFKIYSKHTPINLVRDLPQGFSALEPLVDLPIGINITRFQTLLALHRSYLTPGDSSSGWTAGAAAYYVGYLQPRTFLLKYNENGTITDAVDCALDPLSETKCTLKSFTVEKGIYQTSNFRVQPTESIVRFPNITNLCPFGEVFNATRFASVYAWNRKRISNCVADYSVLYNSASFSTFKCYGVSPTKLNDLCFTNVYADSFVIRGDEVRQIAPGQTGKIADYNYKLPDDFTGCVIAWNSNNLDSKVGGNYNYLYRLFRKSNLKPFERDISTEIYQAGSTPCNGVEGFNCYFPLQSYGFQPTNGVGYQPYRVVVLSFELLHAPATVCGPKKSTNLVKNKCVNFNFNGLTGTGVLTESNKKFLPFQQFGRDIADTTDAVRDPQTLEILDITPCSFGGVSVITPGTNTSNQVAVLYQDVNCTEVPVAIHADQLTPTWRVYSTGSNVFQTRAGCLIGAEHVNNSYECDIPIGAGICASYQTQTNSPRRARSVASQSIIAYTMSLGAENSVAYSNNSIAIPTNFTISVTTEILPVSMTKTSVDCTMYICGDSTECSNLLLQYGSFCTQLNRALTGIAVEQDKNTQEVFAQVKQIYKTPPIKDFGGFNFSQILPDPSKPSKRSFIEDLLFNKVTLADAGFIKQYGDCLGDIAARDLICAQKFNGLTVLPPLLTDEMIAQYTSALLAGTITSGWTFGAGAALQIPFAMQMAYRFNGIGVTQNVLYENQKLIANQFNSAIGKIQDSLSSTASALGKLQDVVNQNAQALNTLVKQLSSNFGAISSVLNDILSRLDPPEAEVQIDRLITGRLQSLQTYVTQQLIRAAEIRASANLAATKMSECVLGQSKRVDFCGKGYHLMSFPQSAPHGVVFLHVTYVPAQEKNFTTAPAICHDGKAHFPREGVFVSNGTHWFVTQRNFYEPQIITTDNTFVSGNCDVVIGIVNNTVYDPLQPELDSFKEELDKYFKNHTSPDVDLGDISGINASVVNIQKEIDRLNEVAKNLNESLIDLQELGKYEQGSGYIPEAPRDGQAYVRKDGEWVLLSTFLGRSLEVLFQGPGHHHHHHHHSAWSHPQFEKGGGSGGGGSGGSAWSHPQFEK

**Figure 9:**
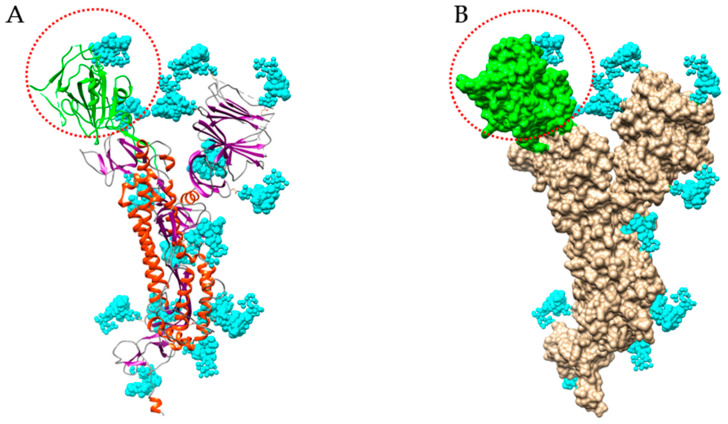
Structure of S-glycoprotein of SARS-CoV-2. (**A**) Ribbon diagram of the heavily glycosylated S-glycoprotein of SARS-CoV-2 (PDB code 6VXX). The RBD bearing 2 *N*-glycans is colored green and circled by a red dotted line. *N*-glycans (biantennary core (GlcNAc)_2_(Man)_5_) are colored cyan. (**B**) Molecular surface representation of the glycosylated S-glycoprotein of SARS-CoV-2. The molecular surface of RBD is colored green.

A detailed study of the *N*- and *O*-glycans attached to the potential *N*- and *O*-glycosylation sites decorating the amino acid sequence of the S-glycoprotein of SARS-CoV-2 (Figure 9), revealed that almost all the putative *N*-glycosylation sites are occupied by a glycan chain, with the exception of the seven potential *N*-glycosylation sites 17NLT, 603NTS, 657NNS, 1134NNT, 1158NHT, 1173NAS and 1194NES. All potential *O*-glycosylation sites T323, S323 and T678, exhibited core-1 type *O*-glycans [25,26].

**Figure 10:**
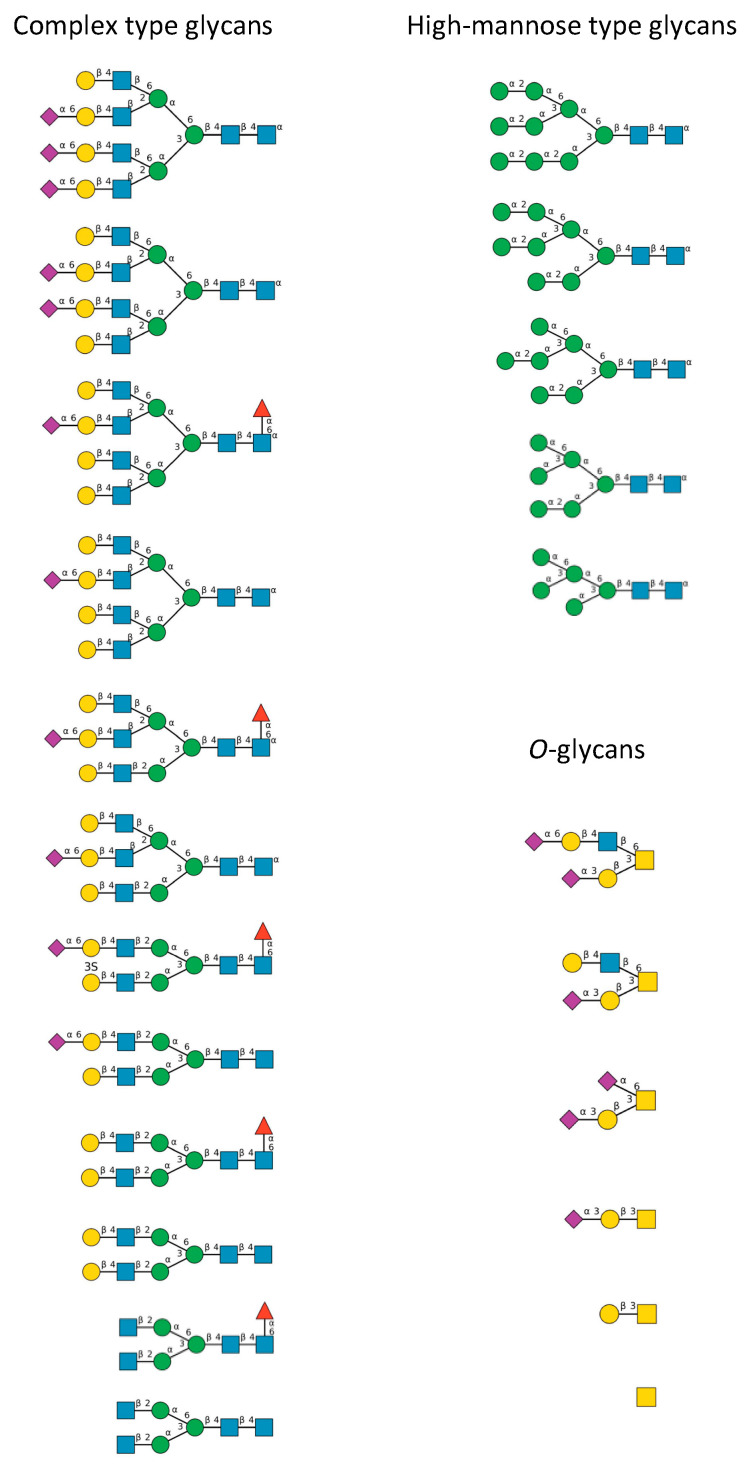
Diversity of the *N*-glycans of the biantennary complex type (left frame) and high-mannose type (upper right frame), and *O*-glycans (lower right frame), identified in the S-glycoprotein forming the spikes at the surface of the SARS-CoV-2 envelope [26]. Symbols used to represent the *N*- and *O*-glycans: blue squares: *N*-acetylglucosamine (GlcNAc), green circles: mannose (Man), yellow circles: galactose (Gal), red triangle: fucose (Fuc), purple diamonds: sialic acid (Neu5Ac), yellow square: *N*-acetylgalactosamine (GalNAc).

A large diversity was observed in the types of *N*-glycans present at the potential *N*-glycosylation sites:

- *N*-glycosylation sites 149NKS, 165NCT, 282NGT, 657NNS, 709NNS, 801NFT, 1074NGT, 1098NGT and 1194NES (often not glycosylated), are almost exclusively occupied by often sialylated, bi-, tri- and tetra-antennary *N*-glycans of the complex type (Figure 10)
- *N*-glycosylation sites 61NVT, 331NIT, 343NAT and 616NCT, are almost exclusively occupied by *N*-glycans of the high-mannose type (Figure 10)
- The remaining *N-*glycosylation 74NGT, 122NAT, 234NIT and 717NFT, contain a mix of *N*-glycans of both types, complex *N*-glycans and high-mannose *N*-glycans (Figure 10)
- Both *O*-glycosylated sites T323, S325, harbor core-1 mucin type *O*-glycans: GalNAc, T-antigen GalNAcGal, sialylated T-antigen GalNAcGalNeuAc_2_, and core-2 sialylated *O*-glycans GalNAcGalNeuAc(GlcNAcGal), and GalNAcGalNeuAc(GlcNAcGalNeuAc) (Figure 10)

Bi- and tri-antennary glycans are predominantly represented, across all categories of the complex *N*-glycans and high-mannose *N*-glycans. Moreover, high-mannose glycans predominantly occur at the top of the S-glycoprotein whereas complex *N*-glycans are localized at the bottom of the glycoprotein, close to the viral envelope surface.

An interesting note is that the RBD (highlighted in green in both the amino acid sequence and tridimensional structure of the S-glycoprotein of SARS-CoV-2), only contains two *N*-glycosylation sites, 331NIT and 343NAT, predominantly occupied by high-mannose *N*-glycans that should be readily accessible to Man-specific seaweed and cyanobacterial lectins.

Most of the complex glycans are sialylated on their Gal residues. Therefore, one can assume that Neu5Ac-specific lectins that have been identified in red algae (Table 3), would recognize the sialylated glycans of SARS-CoV-2. Conversely, the *O*-glycosylation sites T323 and S325, are rather buried at the top of the S-glycoprotein, in such a way that the *O*-glycans are not identified as key targets for the binding of GalNAc/T-Tn-specific lectins to the S-glycoprotein trimer. However, O-glycosylation site T678 is pretty well exposed at the bottom of the S-glycoprotein and therefore, should be accessible to the GalNAc/T-Tn-specific lectins. Accordingly, seaweed lectins with the corresponding specificity should not be relevant glycan probes for SARS-CoV-2, except for the single exposed *O*-glycosylation site T678 (Table 2)

The apparent diversity in glycosylation identified in SARS-CoV-2 and, especially the complex glycans and high-mannose *N*-glycans, has also been reported for the SARS-CoV virus [63], suggesting Man-specific seaweed lectins can be used as glycan probes for other pathogenic coronaviruses.

**Figure 11:**
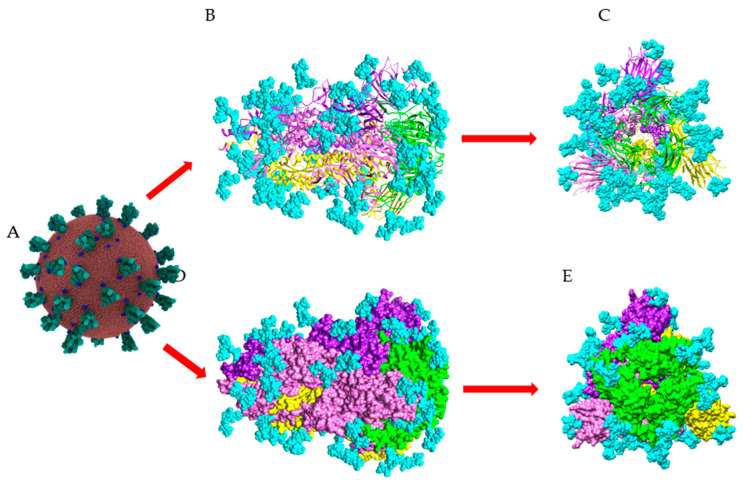
Surface glycosylation of SARS-CoV-2 virus. (**A**) Overall structure of SARS-CoV-2 showing the spikes (colored green) arrayed on the surface of the virus (*Coronavirus* Credit: Maria Voigt/RCSB PDB). (**B**,**D**) Sagital views of the ribbon diagram (**B**) and the molecular surface (**D**), showing the structural organization of the spike (PDB code 6ZGE). The three S-glycoproteins forming the SARS-CoV-2 spike are colored yellow, pink, and purple, respectively. The RBD in each S-glycoprotein is colored green. (**C**,**E**) Front views of the ribbon diagram (**C**) and the molecular surface (**E**), showing the structural organization of the spike. *N*-glycan chains occupying the putative *N*-glycosylation sites in the three S-glycoproteins, are colored cyan and represented in spheres.

The three-dimensional structure of the spike RBD of the S-glycoprotein and the S-glycoprotein monomer, has been solved at atomic resolution, using either X-ray radiocrystallography or cryo- electron microscopy (Table 4). The three-dimensional organization of the spike of SARS-CoV-2 has been also solved in the prefusion conformation, essentially by electron microscopy at lower resolution (Table 4). All these structures confirm the high degree of glycosylation of the S-glycoprotein of SARS-CoV-2 and the lower glycosylation of the RBD, which possesses only two well-exposed sites for *N*-glycosylation (331NIT and 343NAT) and two rather buried sites for *O*-glycosylation (T323 and S325) (Figure 11). In addition to RBD, the highly glycosylated *N*-glycans associated to 234NIT and 282NGT, would play a prominant role in the binding of SARS-CoV-2 to ACE2 receptors of the host cells [25,26,35].

The spikes S-glycoprotein trimers covering the SARS-CoV-2 virions, mediate the binding to the ACE2 receptor through their RBD (S1 subunit), and the subsequent fusion of the viral membrane with the cell membrane (S2 subunit). The spike S-glycoprotein exhibits some flexibility and conformational motions of the S-glycoprotein are pH-dependent [30]. However, neutralizing antibodies readily recognize the closed conformation (all RBD-down) of the spikes, which is highly encouraging for the future development of antibody candidates as potential therapeutic or prophylactic agents (vaccines) against the SARS-CoV-2 [77].

## 4. Man-Specific Seaweed Lectins Interact with *N*-glycans Decorating the S-Glycoprotein from SARS-CoV-2

High-mannose-specific lectins from red algae and BCA from the green alga *Boodlea coacta*, were shown to possess antiviral properties against various enveloped virus including influenza, herpes, and hepatitis C viruses, and HIV-1 (Table 5). In addition, griffithsin exhibited antiviral properties against SARS-CoV coronavirus. Obviously, these antiviral properties depend on the ability of seaweed lectins to specifically recognize and bind high-mannose *N*-glycans that cover the virus envelope.

**Figure 12:**
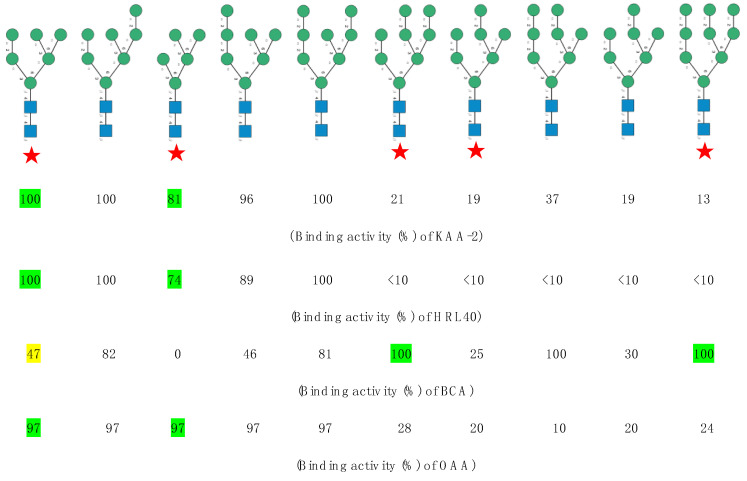
Comparative analysis of the binding activity (expressed as %) of the Man-specific lectins KAA-2 from *Kappaphycus alvarezii*, HLR-40 from *Halimeda renschii*, BCA from the green alga *Boodlea coacta*, and OAA from the blue-green alga (cyanobacterium) OAA from *Oscillatoria agardhii* (adapted from Mu et al. [14] and Sato et al. [11,16]). Symbols used to represent high-mannose glycans: blue squares: N-acetylglucosamine, green circles: mannose. High-mannose glycans identified in the S-glycoprotein of the SARS-CoV-2 are indicated by a red star.

A detailed study of the binding-activity towards pyridylaminated (PA-)-oligosaccharides measured for Man-specific lectins of red algae (KAA-2 from *Kappaphycus alvarezii* [10,13] and HRL-40 from *Halimeda renschii* [14]), green algae (BCA from *Boodlea coacta* [11]) and the cyanobacterium OAA from *Oscillatoria agardhii* [16]), showed that all these lectins readily interact with some of the high mannose *N*-glycans present at the surface of both the influenza virus and HIV-1 envelope, associated to hemagglutinin (influenza virus) or gp120 (HIV-1) glycoproteins (Figure 11). The binding of BCA differs from that measured for other algal lectins due to its preference for the recognition of the Manα1,2 linkage [11]; depending on the number of terminal Manα1,2 that decorate the high-mannose branched glycans of the viral envelope, the binding varies from 0 (*n* = 0) to 100% (*n* = 3) (Figure 12).

An interesting note is that some of the high-mannose glycans of both the hemagglutinin of the influenza virus and the gp120 of HIV-1 recognized by the lectins, also decorate the S-glycoprotein forming the spikes occurring at the surface of the SARS-CoV-2 envelope. Accordingly, the algal Man-specific lectins should similarly interact with the SARS-CoV-2 through the recognition of their spike S-glycoproteins. In this respect, griffithsin (GRFT) was shown to inhibit both the replication and cytopathy of the coronavirus SARS-CoV [36].

**Figure 13:**
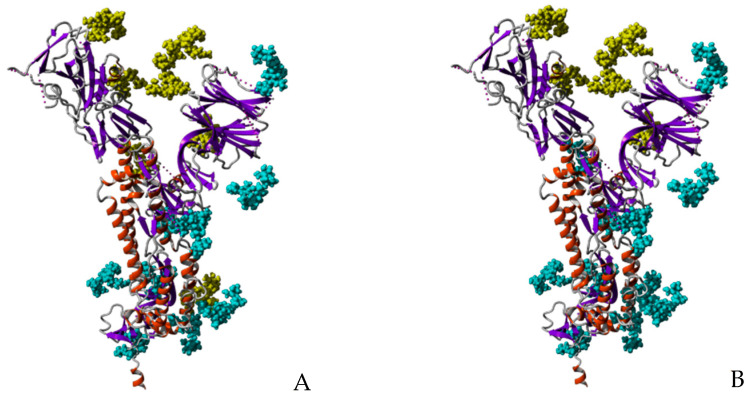
Glycosylation pattern of the monomeric S-glycoprotein of SARS-CoV-2. (**A**) High-mannose type glycans (colored yellow) of the monomeric S-glycoprotein of SARS-CoV-2 susceptible to be specifically recognized by Man-specific lectins KAA-2 and HRL-40 from the red algae *Kappaphycus alvarezii* [10,13] and *Halimeda ronschii* [14], and OAA from the blue-green alga (cyanobacterium) *Oscillatoria agarddhii* [16], are well exposed at the top of the protein. Other complex *N*-glycans decorating the monomer weakly or not recognized by the lectins, are colored cyan. (**B**) High-mannose type glycans (colored yellow) of the monomeric S-glycoprotein of SARS- CoV-2 susceptible to be specifically recognized by the Manα1,2-specific lectin BCA from the green alga *Boodlea coacta* [11]. Other complex *N*-glycans decorating the monomer weakly or not recognized by BCA, are colored cyan.

Looking at the high-mannose type glycans associated to the glycosylation sites N61, N77 (absent from the 3D structure of the S-glycoprotein monomer (PDB code 6VXX)), N122, N234, N331, N343, N616 and N717 occupied by the high-mannose glycans recognized by the Man-specific lectins KAA-2 (*Kappaphycus alvarezii*), HRL-40 (*Halimeda ronschii*), BCA (*Boodlea coacta*) and OAA (*Oscillatoria agadhii*), shows they are the best exposed at the surface of the S-glycoprotein monomer (Figure 13A,B). This is particularly true for the high-mannose glycans specifically recognized by the Manα1,2-specific lectin BCA, which are almost exclusively localized in the external/upper part of the S-glycoprotein monomer (Figure 13B).

**Figure 14:**
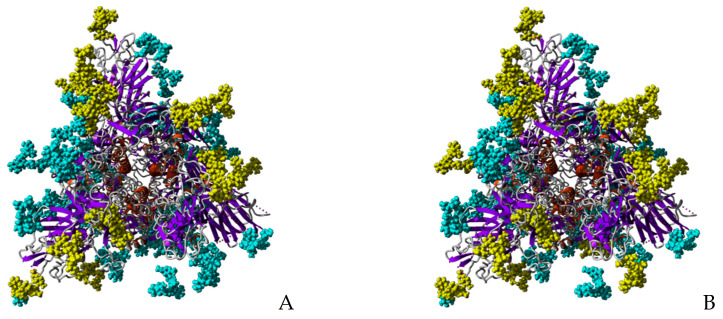
Glycosylation of trimeric S-glycoprotein of SARS-CoV-2. (**A**) Front view of the trimeric S-glycoprotein of SARS-CoV-2 showing the high-mannose type glycans (colored yellow) specifically recognized by Man-specific lectins KAA-2 and HRL-40 from the red algae *Kappaphycus alvarezii* [10,13] and *Halimeda renschii* [14], and OAA from the blue-green alga (cyanobacterium) *Oscillatoria agarddhii* [16]. Other complex *N*-glycans decorating the monomer weakly or not recognized by the lectins, are colored cyan. (**B**) Front view of the trimeric S-glycoprotein of SARS-CoV-2 showing the high-mannose type glycans (colored yellow) specifically recognized by the Manα1,2-specific lectin BCA from the green alga *Boodlea coacta* [11]. Other complex *N*-glycans decorating the monomer weakly or not recognized by BCA, are colored cyan.

A front view of the trimeric S-glycoprotein of SARS-CoV-2 clearly shows that most of the high-mannose recognized by lectins KAA-2, HRL-40, BCA and OAA, are nicely exposed at the surface of the trimer and are thus readily available for interacting with Man-specific seaweed lectins (Figure 14).

## 5. Interaction of Other Seaweed Lectins with Different Specificities with the S-Glycoprotein from SARS-CoV-2

**Figure 15:**
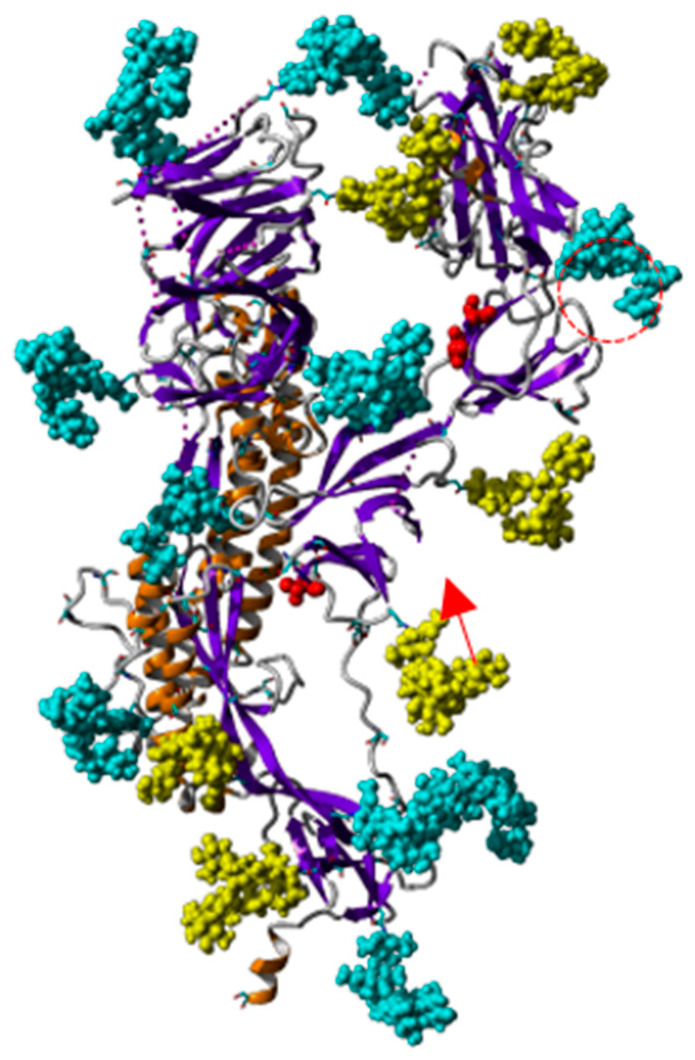
Ribbon diagram of the monomeric S-glycoprotein of SARS-CoV-2 showing the buried character of the *O*-glycosylated T323 and S325 amino acid residues (red dashed circle). High-mannose type glycans recognized by Man-specific seaweed lectins are colored yellow. Another exposed *O*-glycosylated T678 occurs in the S-glycoprotein (red arrow). Other often sialylated complex type *N*-glycans, well exposed at the surface of the S-glycoprotein monomer, are colored blue.

Due to the diversity of the glycans decorating the S-glycoprotein of SARS-CoV-2, namely (sialylated) *N*-glycans of the complex type and *O*-glycans, seaweed lectins with different specificities should bind to the spikes covering the viral envelope. In this respect, seaweed lectins that specifically recognize GalNAc and the T/Tn antigens (*O*-glycans) and seaweed lectins specific for terminal Neu5Ac residues, could interact with the S-glycoprotein of SARS-CoV-2 (Table 2). Looking at the localization of both types of glycans at the surface of the S-glycoprotein, shows that *O*-glycans are attached to the rather buried T323 and S325 residues of S-glycoprotein, that most probably prevents their recognition by GalNAc/T/Tn-specific seaweed lectins [25]. However, another more exposed *O*-glycosylation site has been identified at T678 [26] (Figure 15). Thus, with the exception of *O*-glycan site T678 that could be recognized by GalNAc/T-Tn-specific seaweed lectins, only Neu5Ac-specific seaweed lectins could readily interact with the sialylated complex type *N*-glycans well exposed at the surface of the S-glycoprotein, even though most of them are located at the bottom of the S-glycoprotein and thus, are less accessible to the lectins.

## 6. Bioinformatics

The atomic coordinates of griffithsin GRFT from *Griffithsia* sp., including the unliganded lectin (PDB code 2GTY) [87], and lectin complexed to mannose (PDB code 2GUD) [87], 6α-mannobiose (PDB code 2HYQ) [88] and high-mannose branched carbohydrate (PDB code 3LL2) [89], were taken from the Protein Data Bank PDB (http://www.rcsb.org/pdb/) [90]. Similarly, the atomic coordinates of the unliganded and complexed to α3, α6-mannopentaose forms of the cyanobacterial Man-specific lectin BOA from *Burkholderia oklahomensis* (PDB code 4GU8 and 4GK9) [91] and the unliganded form of OAA from *Oscillatoria agardhii* (PDB code 3OBL) [92] were also obtained from the PDB.

Homology modeling of other lectins including the β-prism II folded GCL from *Grateloupia chiangii* [15], the β-barrel folded KAA-2 from *Kappaphycus alva*rezii [10], and the β-sandwich folded NgL from *Porphyra umbilicalis* [44], was performed with the YASARA Structure program [93] using various protein templates from the PDB, depending on the overall structural scaffold to which they belong. PROCHECK [94], ANOLEA [95], and the calculated QMEAN scores [96,97], were used to assess the geometric and thermodynamic qualities of the three-dimensional models.

Docking of simple sugars and oligosaccharides was performed with YASARA and SwissDock [98]. Hydrophilic/hydrophobic regions at the surface of the lectins were calculated and displayed with Chimera [99]. Molecular cartoons were drawn with Chimera [99] and YASARA [93].

## 7. Discussion

The S-glycoprotein on the surface of the SARS-CoV-2 virus is a highly glycosylated protein. Due to the exposed localization of high-mannose glycans at the top of the S-glycoprotein trimers many of these glycans are readily accessible to carbohydrate-binding proteins. Seaweed lectins represent well adapted glycan probes for the specific recognition of this type of viruses. In this respect, the Man-specific lectin griffithsin (GRFT) of the red alga *Griffithsia* sp., readily recognized the high mannose *N*-glycans located on the very similarly glycosylated SARS-CoV S-glyco- protein [9,63]. More generally, in agreement with their capacity to specifically recognize high- mannose glycoprotein targets exposed at the surface of enveloped viruses, e.g., hemagglutinin of influenza virus, gp120 of HIV-1 or the spike S-glycoprotein of SARS-CoV and SARS-CoV-2, Man-specific seaweed lectins can interfere with the mechanisms allowing the infectious viruses to recognize the corresponding receptors and trigger the fusion events necessary for entering the susceptible cells. As previously reported [9], GRFT was shown to inhibit both the replication and cytopathy of the closely-related coronavirus SARS-CoV. Accordingly, other Man-specific seaweed lectins could act as blockers, at least in vitro, of the replication for the SARS-CoV-2 virus, and display antiviral properties as already shown for cyanobacterial Man-specific lectins towards a broad range of enveloped viruses including influenza virus, Ebola virus, herpes virus, hepatitis C virus and HIV-1.

Moreover, the binding of seaweed lectins to SARS-CoV-2 virus could be applied in biomedical research, e.g., using Man-specific seaweed lectins (1) for detection purposes of the virus on various contaminated surfaces such as doorknobs or furniture elements, (2) as an efficient barrier to avoid the shedding into the environment of contaminating virions and, (3) as control reagents for the occurrence of viral particles in biotic/abiotic samples. Depending on the case, whether properly labelled, e.g., fluorochrome-labelled, Man-specific seaweed lectins could be used directly as glycan probes or unlabelled lectins could be further detected using properly labelled, e.g., fluorochrome-labelled, specific anti-lectin antibodies.

The antiviral properties of Man-specific seaweed lectins and the application of these lectins as blocking agents for the replication of enveloped viruses still requires more investigation. So far, the antiviral properties of Man-specific seaweed lectins, have been demonstrated essentially in in vitro conditions (Table 5). Indeed, only few studies have shown to block the replication of SARS-CoV and other coronaviruses in vivo [100]. O’Keefe et al. (2010) reported on the use of GRFT to prevent the SARS-CoV infection both in vitro and in vivo, and showed that GRFT treatment reduces mortality and morbidity in a lethal infection mouse model [100]. Millet et al., (2016), further pointed out the inhibitory effect of GRFT towards Middle East respiratory syndrome coronavirus MERS-CoV [101] Time-course experiments revealed that GRFT inhibits MERS-CoV infection at the early step when the virus binds the host cells. Next to seaweed lectins, closely related plant lectins with different carbohydrate-binding specificities have been investigated in vitro for their antiviral activity against SARS-CoV and another coronavirus FIPV, responsible for feline infectious peritonitis [102]. Although plant lectins specific for Gal, GalNAc and GlcNAc, exhibited some antiviral activity, a much higher antiviral activity towards both coronaviruses was reported especially for Man-specific lectins belonging to the family of GNA-related lectins, such as GNA from *Galanthus nivalis* (snowdrop), NPA from *Narcissus pseudonarcissus* (daffodil) and APA from *Allium porrum* (leek). In addition, two targets for these Man-specific lectins in the replication cycle of SARS-CoV have been identified, one in the early phase of the replication cycle during viral attachment, and a second target at the end of the infection cycle [102]. More recently, the lectin FRIL from hyacinth bean (*Lablab purpureus*), which specifically recognizes *N*-glycans of the complex type occurring on the surface of coronavirus envelope, was demonstrated to neutralize SARS-CoV-2 and prevent both viral protein production and cytopathic effects in host (mice) cells [90].

At the molecular level, the mechanism of action for Man-specific lectins is primarily referred to a masking effect of the molecular surface of S-glycoprotein RBDs due to their interaction with the Man-containing glycans, thus hampering the proper attachment of the virions to the host cell receptors and preventing the viral replication. However, the identification of a second target for HHA, the Man-specific lectin from *Hippeastrum hybridum*, at the end of the SARS-CoV infection cycle [102], suggests that Man-specific lectins interfere not only with the virus entry in the host cells but also with the virus release from the host cells. The spike S-glycoprotein most probably is the main glycan target for Man-specific lectins but the specific recognition of other Man-containing targets cannot be excluded. In this respect, the heavily glycosylated ACE2 receptor could also serve as relevant target for Man-specific lectins.

Although lectins remain attractive anti-coronavirus candidates, at present it remains difficult to correctly assess the actual role of these natural compounds in the therapeutic armamentarium, to fight against SARS-CoV-2, the coronavirus responsible for the highly transmissible infectious COVID-19 [103,104,105,106].

## Figures and Tables

**Table 1:** List of Man-specific seaweed lectins.

Seaweed Family	Seaweed Species	Lectin	Structural Scaffold	Ref.
Red algae	*Agardhiella subulata*	ASL-1,	β-barrel	[7]
		ASL-2	β-barrel	
	*Carpopeltis flabellata*	CFA	unknown	[36]
	*Eucheuma amakusaensis*	EAA-1	β-barrel	[37]
		EAA-2	β-barrel	
		EAA-3	β-barrel	
	*Eucheuma cottonii*	ECA-1	β-barrel	[37]
		ECA-2	β-barrel	
	*Eucheuma denticulatum*	EDA-1	β-barrel	[38]
		EDA-2	β-barrel	
	*Eucheuma serra*	ESA-1	β-barrel	[39,40]
		ESA-2	β-barrel	
	*Gracilaria bursa-pastoris*	GBPL	unknown	[41]
	*Grateloupia chiangii*	GCL	β-prism II	[15]
	*Griffthsia* sp.	griffithsin	β-barrel	[8]
	*Kappaphycus alvarezii*	KAA-2	β-barrel	[10]
	*Kappaphycus striatum*	KSA-2	β-barrel	[38]
	*Meristiella echinocarpa*	MEL	β-barrel	[42]
	*Meristotheca papulosa*	MPA-1	β-barrel	[42]
		MPA-2	β-barrel	
	*Solieria filiformis*	SfL-1	β-barrel	[12]
		SfL-2	β-barrel	
	*Solieria robusta*	SrL	β-barrel	[3]
	*Hydropuntia (Gracilaria) fisheri*	HFA	β-sandwich	[43]
	*Porphyra umbilicalis*	NgL	β-sandwich	[44]
Yellow-green algae	*Nannochloropsis gaditana*	BU14	β-sandwich	[45]
Green algae	*Boodlea coacta*	BCA	β-prism II	[11,46]
	*Bryopsis plumosa*	BPL-2	MFP2-like scaffold	[47]
	*Enteromorpha prolifera*	EPL-1/2	unknown	[48]
	*Halimeda renschii*	HRL40-1/2	unknown	[14]
	*Ostreococcus tauri*	OtL	β-sandwich	[49]

**Table 2:** List of GalNAc/T/Tn-specific seaweed lectins.

Seaweed Family	Seaweed Species	Lectin	Structural Scaffold	Ref.
Rhodophyceae	*Aglaothamnion oosumiense*	AOL1	unknown	[56]
Chlorophyceae	*Codium fragile*	CFL	unknown	[57,58]

**Table 3:** List of Neu5Ac-specific seaweed lectins.

Seaweed Family	Seaweed Species	Lectin	Structural Scaffold	Ref.
Rhodophyceae	*Gracilaria tikvahiae*	GTL	unknown	[59]
	*Palmaria palmata*	PPL	unknown	[60]
	*Solieria chordalis*	ScL	β-barrel	[61]
Phaeophyceae	*Fucus vesiculosus*	? *	unknown	[62]

* The protein nature of the so-called *Fucus vesiculosus* lectin needs further confirmation.

**Table 4:** List of the RBD (Receptor-binding domain) and S-GPT (S-Glycoprotein trimer) solved by either X-ray radiocrystallography and/or cryo-electron miscroscopy (Cryo-Em). Tbp: to be published (atomic coordinates available at the PDB but results are unpublished by the authors).

RBD/S-GPT	PDB Code	RX/Cryo-Em	Resolution (Å)	Ref.
RBD	6W41	RX	3.084 Å	[64]
RBD	6XC2, 6XC3, 6XC4, 6XC7	RX	2.3 Å–3.11 Å	[65]
RBD	6XDG	Cryo-Em	3.9 Å	[66]
RBD	6XE1	RX	2.75 Å	[67]
RBD	6YLA, 6YM0, 6YOM	RX, Cryo-Em	2.42 Å–4.36 Å	Tbp
RBD	6YZ7, 6Z2M, 6ZH9	RX	2.71 Å–3.31 Å	Tbp
RBD	6ZCZ, 6ZER, 6ZFO	RX, Cryo-Em	2.65 Å–4.4 Å	[68]
RBD	7BWJ	RX	2.85 Å	[69]
RBD	7BZ5	RX	1.84 Å	[70]
RBD	7C01	RX	2.88 Å	[28]
RBD	7C8V	RX	2.15 Å	Tbp
RBD	7JMP	RX	1.712 Å	[71]
S-GPT	6VYB	Cryo-Em	3.2 Å	[72]
S-GPT	6WPT	Cryo-Em	3.1 Å–3.7 Å	[73]
S-GPT	6X2A	Cryo-Em	2.9 Å–3.6 Å	[74]
S-GPT	6X6P	Cryo-Em	3.22 Å	[29]
S-GPT	6X79	Cryo-Em	2.9 Å	[75]
S-GPT	6XCN	Cryo-Em	3.42 Å–3.66 Å	[76]
S-GPT	6XEY	Cryo-Em	3.27 Å	[77]
S-GPT	6XF5,6XF6	Cryo-Em	3.45 Å–4.0 Å	Tbp
S-GPT	6XKL	Cryo-Em	3.21 Å	[78]
S-GPT	6XLU,6XM0,6XM3,6XM4,6XM5	Cryo-Em	2.4 Å–3.1 Å	[31]
S-GPT	6XR8	Cryo-Em	2.9 Å	[79]
S-GPT	6XS6	Cryo-Em	3.7 Å	[80]
S-GPT	6Z43	Cryo-Em	3.3 Å	Tbp
S-GPT	6Z97	Cryo-Em	3.4 Å	[81]
S-GPT	6ZDH	Cryo-Em	3.7 Å	[68]
S-GPT	6ZGE,6ZGH,6ZGG,6ZGI,6ZHD	Cryo-Em	2.6 Å–6.8 Å	Tbp
S-GPT	6ZOX,6ZOY,6ZOZ,6ZP0,6ZP1,6ZP2	Cryo-Em	3.0 Å–3.5 Å	[29]
S-GPT	6ZOW,6ZP5, 6ZP7	Cryo-Em	3.0 Å–3.3 Å	[82]
S-GPT	6ZWV	Cryo-Em	3.5 Å	[83]
S-GPT	7BYR	Cryo-Em	3.84 Å	[84]
S-GPT	7CN9	Cryo-Em	4.7 Å	[85]
S-GPT	7JJI	Cryo-Em	3.6 Å	[86]

**Table 5:** List of seaweed lectins tested for their antiviral properties against enveloped viruses possessing exposed glycans.

Seaweed Family	Lectin	Virus	Glycan Recognized	Ref.
Rhodophyceae	ESA-2 (*Eucheuma serra*)	Influenza	High-mannose	[12]
	GCL (*Grateloupia chiangii*)	Influenza	High-mannose	[15]
		Herpes		
	Griffithsin (*Griffithsia* sp.)	HIV-1	High-mannose	[8]
		Hepatitis C		
		SARS-CoV		[9]
	HRL-40 (*Halimeda renschii)*)	Influenza	High-mannose	[14]
	KAA-2 (*Kappaphycus alvarezii*)	Influenza	High-mannose	[10]
		HIV-1		[13]
Chlorophyceae	BCA (*Boodlea coacta*)	Influenza	High-mannose	[11]
		HIV-1		
* Cyanobacteria	MVN (*Microcystis aeruginosa*)	HIV-1	High-mannose	[22]
	MVL (*Microcystis viridis*)	HIV-1	High-mannose	[23]
	CV-N (*Nostoc ellipsosprum*)	HIV-1	High-mannose	[17,18]
		Herpes		[18]
		Ebola		[19]
		Hepatitis C		[20]
		Influenza		[21]
	OAA (*Oscillatoria agardhii*)	HIV-1	High-mannose	[16]
	SVN (*Scytonema varium*)	HIV-1	High-mannose	[24]

* Cyanobacteria lectins with antiviral properties were included.

## Abbreviations

AOL1	*Aglaothamnion oosumiense* lectin
APA	*Allium porrum* agglutinin
ASL	*Agardhiella subulata* lectin
BCA	*Boodlea coacta* lectin
BOA	*Burkholderia oklahomensis* agglutinin
BPL2	Lectin 2 of *Bryopsis plumosa*
BU14	*Nannochloropsis gaditana* lectin
CBS	Carbohydrate-binding site
CFA	*Carpopeltis flabellata* agglutinin
CFL	*Codium fragile* lectin
CV-N	Cyanovirin-N (*Nostoc ellipsosporum*)
EEA	*Eucheuma amakusanensis* lectin
ECA	*Eucheuma cottonii* lectin
EDA	*Eucheuma denticulatum* lectin
EPL	*Enteromorpha prolifera* lectin
ESA	*Eucheuma serra* lectin
FRIL	Flt3 receptor interacting lectin from hyacinth bean (*Lablab purpureus*)
Fuc	Fucose
Gal	Galactose
GalNAc	*N*-acetylgalactosamine
GBPL	*Gracilaria bursa-pastoris* lectin
GCL	*Grateloupia chiangii* lectin
GlcNAc	*N*-acetylglucosamine
GNA	*Galanthus nivalis* (snowdrop) agglutinin
GPT	Glycoprotein trimer
GRFT	Griffithsin
GTL	*Gracilaria tikvahiae* lectin
HFA	*Hydropuntia* (*Gracilaria*) *fisheri* agglutinin
HIV-1	Human Immunodeficiency Virus
HRL40	*Halimeda renschii* lectin
KAA-2	Lectin 2 of *Kappaphycus alvarezii*
KSA-2	Lectin 2 of *Kappaphycus striatum*
Man	Mannose
MEL	*Meristiella echinocarpa* lectin
MPL	*Meristotheca papulosa* agglutinin
MVL	*Microcystis viridis* lectin
MVN	Microvirin (*Microcystis aeruginisa*)
Neu5Ac	N-acetylneuraminic acid (sialic acid)
OAA	*Oscillatoria agardhii* lectin
OtL	*Ostreococcus tauri* lectin
PDB	Protein Data Bank
PPL	*Palmaria palmata* lectin
PUL	*Porphyra umbilicalis* lectin
RBD	Receptor binding domain
SARS-CoV-2	Severe acute respiratory syndrome-coronavirus
Sia	Sialic acid
ScL	*Solieria chordalis* lectin
SfL	*Solieria filiformis* lectin
SrL	*Solieria robusta* lectin
SVN	Scytovirin (*Scytonema varium*)
T/Tn	T and Tn antigens (Thomsen Friedenreich antigens)
